# Therapeutic Peptides Are Preferentially Solubilized in Specific Microenvironments within PEG–PLGA Polymer Nanoparticles

**DOI:** 10.1021/acs.nanolett.3c04558

**Published:** 2024-02-02

**Authors:** Raquel López-Rios de Castro, Robert M. Ziolek, Martin B. Ulmschneider, Christian D. Lorenz

**Affiliations:** † Department of Chemistry, King’s College London, London SE1 1DB, United Kingdom; ‡ Biological Physics and Soft Matter Group, Department of Physics, King’s College London, London WC2R 2LS, United Kingdom; ¶ Kvantify Aps, DK-2300 Copenhagen S, Denmark

**Keywords:** Molecular dynamics simulations, PEG, PLGA, polymer nanoparticles, drug delivery vehicles

## Abstract

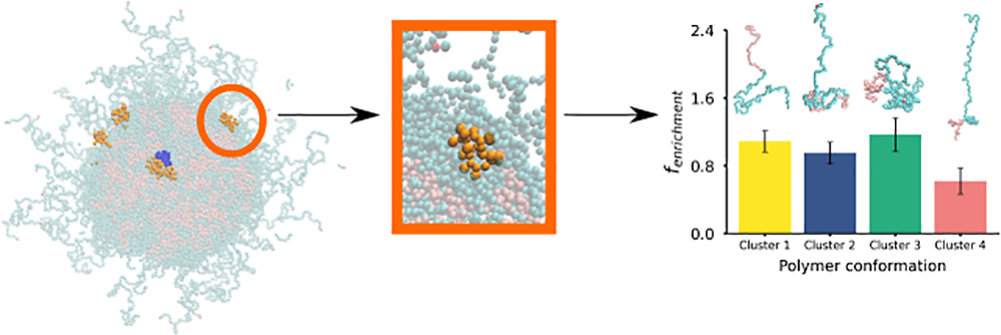

Polymeric nanoparticles are a highly promising drug delivery formulation. However, a lack of understanding of the molecular mechanisms that underlie their drug solubilization and controlled release capabilities has hindered the efficient clinical translation of such technologies. Polyethylene glycol-poly(lactic-*co*-glycolic) acid (PEG–PLGA) nanoparticles have been widely studied as cancer drug delivery vehicles. In this letter, we use unbiased coarse-grained molecular dynamics simulations to model the self-assembly of a PEG–PLGA nanoparticle and its solubulization of the anticancer peptide, EEK, with good agreement with previously reported experimental structural data. We applied unsupervised machine learning techniques to quantify the conformations that polymers adopt at various locations within the nanoparticle. We find that the local microenvironments formed by the various polymer conformations promote preferential EEK solubilization within specific regions of the NP. This demonstrates that these microenvironments are key in controlling drug storage locations within nanoparticles, supporting the rational design of nanoparticles for therapeutic applications.

In the last 30 years, nanomedicine and, in particular, drug-loaded polymeric nanoparticles (NPs),^1,2^ have attracted significant attention as potential candidates for improving therapeutic delivery, including tackling cancer.^1^ Polymer-based NPs have several characteristics that make them ideal delivery vehicles for cancer therapeutics, such as the biodegradability of their polymeric components,^3^ increased circulation time of the encapsulated drug,^4,5^ and a high NP drug-loading capacity.^6,7^ In particular, poly(ethylene glycol)–poly(lactic-*co*-glycolic) acid (PEG–PLGA) NPs have been the subject of numerous studies, as they have been shown to successfully deliver anticancer drugs to tumorous tissues *in vitro*(6,8) and in animal models.^9,10^

PEG–PLGA block copolymers are amphiphilic, self-assembling into core–shell nanoparticles with significant drug encapsulation potential.^11−13^ PEG, which principally forms the hydrophilic corona of these NPs, increases the water solubility of the NPs, leading to an increased circulation lifetime and reduced toxicity.^14,15^ Furthermore, PEG-coated NPs have a significantly reduced systemic clearance compared to non-PEG NPs.^9^ On the other hand, the PLGA blocks form the hydrophobic core of these PEG–PLGA NPs. PLGA plays an important role in controlled drug release,^9^ reduces the cellular uptake of the NP by healthy cells via the endocytic route, and increases the drug circulation time *in vivo*.^16^ Also, due to their amphiphilic nature, PEG–PLGA NPs can encapsulate drugs with low water-solubility encapsulated in the PLGA core,^15^ and hydrophilic drugs within the PEG corona.^17^

While PEG–PLGA NPs have been shown to be successful at encapsulating a range of small-molecule therapeutics and delivering them to cancer cells *in vitro* and in mouse models, the lack of a clear understanding of the molecular mechanisms that govern the structure of these NPs, their ability to encapsulate small molecules, and their interactions with cells has prohibited them from having similar success in clinical applications.^18,19^ These processes are highly dynamic and challenging to study experimentally; however, a deep understanding at the molecular level is possible with molecular dynamics (MD) simulations. For example, Stipa et al.^12^ have used all-atom MD simulations to study how PLGA and PLA NPs interact with paracetamol, prednisolone, and isoniazid. However, in this work, the drugs were randomly added to the NPs, so the encapsulation process of the drugs was not captured. Most literature focuses only on small PLGA NPs^17,20^ or simplified models consisting of only one drug molecule and a very small number of polymers.^13,21^ Since the polymer species, length, and concentration will affect the self-assembly, structure, and physicochemical characteristics of the nanoparticle, a molecular understanding of how experimentally validated PEG–PLGA NPs self-assemble and encapsulate their cargo is needed.

In this work we used unbiased coarse-grained (CG) MD simulations to investigate the self-assembly of a PEG–PLGA NP and the simultaneous solubilization of the anticancer peptide EEK. This exact formulation has been tested against triple-negative breast cancer cells *in vivo*.^22^ From our simulation, we study the internal structure of the NP and, correspondingly, the local environment of EEK within the NP. We provide a molecular-level understanding of PEG–PLGA nanoparticle cargo loading by finding specific polymer conformations that drive the solubilization of EEK.

We performed a CG MD simulation of the self-assembly of the PEG–PLGA NP along with EEK using GROMACS 2020.3,^23^ with the MARTINI (martini22p) force field.^24^ The MARTINI force field has been widely used to simulate PEG^25,26^ and PLGA^27^ polymers and α-helical proteins,^28^ which are the cargo encapsulated in the NP studied in this letter. The polarizable water model more accurately represents an aqueous solution^29^ and the martini22p force field has been previously shown to accurately model PEG;^30^ a nonstandard MARTINI topology, which includes PEG,^31^ was used. The atomistic structure of the peptide, EEK, was converted to a CG representation using the CHARMM-GUI Martini Solution Maker.^32,33^ Previously reported experiments used a molar ratio of EEK to PEG–PLGA (PEG average *M*_*n*_ = 5000, PLGA *M*_*n*_ = 7000, and a PLA:PGA ratio of 1:1) of 1:14 (weight ratio is 1:100).^22^ As well as reproducing the experimental synthesis procedure within the simulation protocol, we have the same molecular weight polymer, polymer concentration, and PEG:PLGA ratio as were used in the experiments. 200 PEG–PLGA copolymers and 15 EEK peptides were randomly placed within a box of water with dimensions of 20 × 20 × 20 nm^3^ (the system consists of 600 417 CG beads). This system is subsequently inserted into a larger box with a size of 58 × 58 × 58 nm^3^ so that the various components have space to assemble into the NP. Polarizable MARTINI water was added to the system, and sodium (Na^+^) and chlorine (Cl^–^) ions were added such that the salt concentration is 0.15 M. Periodic boundary conditions were used. The long-range electrostatic interactions were computed using the reaction field algorithm^34^ with the cutoff distance set to 11 Å. Lennard-Jones interactions also have a cutoff distance of 11 Å. Two consecutive steepest-descent minimizations were performed to remove any undesirable steric artifacts. These energy minimizations were followed by a temperature equilibration simulation in the NVT ensemble (4 ns) at 303.15 K, using the leapfrog integrator with a 20 fs time step. The velocity-rescale thermostat was used,^35^ with separate thermostat groups assigned to the EEK molecules, PEG–PLGA polymers, ions, and water molecules. The production simulation was performed in the NPT ensemble at 350 K and at a pressure of 1 atm for 935 ns using the Parrinello–Rahman barostat (all other simulation details are the same as for the NVT run).^36^

In order to determine when the NP structure had reached stationarity, we considered the time evolution of the fraction of PLGA monomers found instantaneously within different regions of the NP core. This methodology has previously been used to study Pluronic and Tetronic micelles.^37^Figure S1 shows that these quantities become stationary at approximately 0.6 μs, implying that the internal structure of the NP has reached equilibrium at that point. Figure S2 shows the radius of the NP (and its core alone) over time; we note that these values plateau more quickly (after approximately 0.3 μs). Therefore, it is important to consider the internal structure of equilibrating micelles, rather than just their bulk size, in order to accurately quantify structural equilibration in MD simulations. The analysis presented in this letter was performed using only the stationary portion of the micelle trajectory; i.e., a burn-in time of 0.6 μs was used (so only the final 400 ns of the simulation trajectory was considered in the analysis). The average radius of gyration of the NP at equilibrium (9.93 ± 0.06 nm) shows good agreement with the experimentally measured diameter (20 nm) for this formulation.^22^

To understand how the polymer blocks and peptides are distributed within the NP, we calculated the radial density of NP components (Figure 1(a)). The NP core is predominantly made up of LA and GA blocks. Surprisingly, a small nucleus of water forms close to the center of the NP core during the self-assembly process in the unbiased CG MD simulation (this is indicated in Figure 1(a) by a peak in the water density curve (dark blue) centered at *r* ≈ −70 Å). In this central region, there are also peaks in the density of both PEG and EEK. The EEK peptides are found at the interface of the small nucleus of water, as highlighted in the snapshot (Figure 1(c)). To quantify the amount of each polymer species in the core of the NP, Figure 1(b) shows the percentage of coarse-grained beads representing the polymer species and water within the core. PLGA blocks account for 70% of the total number of beads within the hydrophobic core, with water making up 1.4% of the core beads. The EO density peak at *r* ≈ 5 Å (and the sudden decrease in the density of the LA and GA monomers) denotes the boundary between the NP core and its hydrophilic PEG-based corona. There is also a peak in the EEK density at the core–shell interface at *r* ≈ 0 Å, showing that peptides are located at the core–corona interface (as well as in the NP core). We determined the distance between the center of mass (COM) of each EEK and the COM of the NP over time during the stationary part of the simulation (Figure S4); two EEK molecules are encapsulated within the core of the NP throughout this part of the simulation, while the remaining EEK molecules are found at the core–shell interface. We do not observe transport of EEK within the NP after the NP self-assembly and NP structural equilibration portions of the simulation conclude.

**Figure 1:**
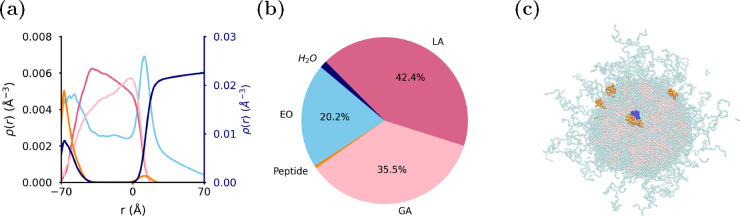
Internal composition of the PLGA–PEG nanoformulation. (a) Spherical density of various components of the polymeric NP and its aqueous environment, where glycolic acid (GA) is shown in light pink, lactic acid (LA) in fucsia, EO in light blue, EEK in orange, and water in navy blue. (b) Percentage of the NP core made up by each polymer block, EEK (“peptide”), and water. EEK peptides account for 0.5% of the beads in the core. (c) Snapshot of the cross section of the polymeric NP loaded with EEK peptides, where PLGA is shown in pink, PEG in light blue, peptide in orange, and water in navy blue. The two peptides located in the inner core can be clearly seen in the middle of the NP core. This representation is not to scale.

To determine the mechanisms of peptide encapsulation in the two different locations within the NP, we investigated the interactions of the EEK molecules with the polymer blocks and water (Figure 2, see SI for full methodology details). Figure 2(a) shows that the EEK peptides inside the core are generally less hydrated than the peptides on the surface, as expected. As we detailed previously, there is a small amount of water close to the NP core center, so the peptides within the core do still interact with water. The polymer blocks do not completely shield EEK from water, in agreement with previous computational studies of peptide solubilization by polymers.^13^Figure 2(b) shows the difference in contacts between EEK and all polymer beads at the core center and the core–corona interface. In the core center, most peptide residues exhibit a greater number of contacts with polymers than those peptides at the core–shell interface, as might reasonably be expected. ALA and LYS residues of the core residing peptides have the fewest polymer contacts but are the most heavily hydrated. So for peptides in the core center, these residues are most commonly found to be in contact with the small water nucleus in the NP core.

**Figure 2:**
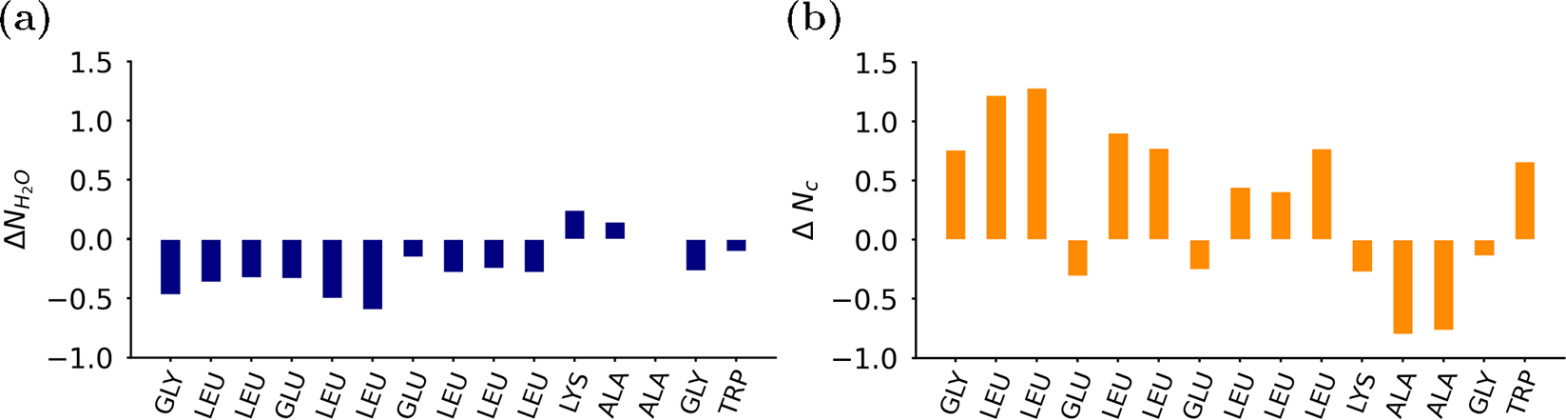
Time-averaged cargo contacts and the hydration difference between peptide storage locations. (a) Difference between the time-averaged water–EEK contacts (hydration) per amino acid between the peptides within the core and at the core–corona interface. (b) Difference between the time-averaged polymer–EEK contacts per amino acid between the peptides within the core and at the core–corona interface. A positive value corresponds to more contacts between a specific EEK residue and either a polymer bead or water in the center of the core than at the core–corona interface and vice versa for a negative value.

Table 1 shows the time-averaged enrichment of contacts between the peptides in the two different locations and for each of the polymer species. The peptides have higher interactions with PEG in both locations. However, it is interesting to note that the peptides located in the core have a much higher tendency to interact with EO monomers than the peptides located at the core–shell interface. We attribute this to the amphiphilicity of EEK, resulting in its close proximity to the trapped water in the NP core. EO is hydrophilic, and surrounding the water in the core is a peak in EO density (Figure 1(a)). EO is found around the water trapped in the core, resulting in a larger number of interactions between EEK and EO within the NP core.

**Table 1:** Contact Enrichment between EEK Peptides and Polymer Species^a^

Location	LA	GA	EO
Core center	0.33 [0.32, 0.34]	0.37 [0.36, 0.39]	2.19 [2.17, 2.21]
Core–shell interface	0.48 [0.47, 0.48]	0.30 [0.29, 0.30]	1.59 [1.58, 1.59]

a Contact enrichment quantifies the extent to which one molecule exhibits a higher tendency to interact with another molecule, relative to their respective abundances. This table shows the contact enrichment between polymer species and EEK peptides, differentiating between the two storage location: the core and the core–shell interface. In this case, the enrichment takes into account the polymer species and peptide population in the areas of cargo encapsulation. A value greater than 1 means a tendency to interact with that species, 1 indicates no preference in interactions, and a value of less than 1 is the tendency to not interact with that polymer species. Values reported are the contact enrichment average over time with its corresponding 90% CI in square brackets. Further information about this method can be found in the SI.

To provide an assessment of the dynamics of the peptides within the NP, we consider the time evolution of each peptide’s local environment (defining the instantaneous local environment as the polymer beads found within a cutoff distance of the peptide at a given time). We calculated the autocorrelation function (ACF) of the local environment (see Figure S5). Both the peptides in the core and those at the core–corona interface readily exchange between different polymer beads, indicating that they are not static within the NP; the local environment of the two peptides in the core of the NP changes more slowly than those peptides at the core–corona interface, showing that they are less dynamic, as might be expected.

To investigate the specific conformations that the polymers adopt within the NP, we applied a two-step unsupervised machine learning protocol consisting of a dimensionality reduction with UMAP and clustering in the subsequent embedding using HDBSCAN (for the full methodology, see the SI section “Dimensionality reduction and clustering”).^37−39^Figure 3(a) shows the four clusters that were identified via this procedure (0.41% of molecular conformations were not clustered). Each cluster groups together polymers of a similar conformation. The polymer conformation is defined by the end-to-end distances of both the PLGA and the PEG blocks. Cluster 1, the most frequently identified conformation, has a collapsed PEG block and an extended PLGA block. Cluster 2 possesses an extended PEG block and a collapsed PLGA block, while in cluster 3, both blocks are collapsed. Cluster 4, the least frequently observed conformation, has a particularly extended EO block as well as an extended PLGA block. Each cluster therefore represents different polymer conformations, which can be readily understood in a physical sense. The embedded space and the average block distances of each cluster are shown in Figure S6. We then calculated the intrinsic density of each conformational cluster (Figure 3(b)) to reveal the spatial distribution of each conformation within the NP, with respect to the core–corona interface. We note that cluster 1, with its extended PLGA block, exhibits a high density in the center of the NP core. Cluster 4, which conversely has a particularly extended PEG block, is surprisingly also found at high density in the NP core center: the small nucleus of water at the NP promotes the unexpected location of this conformational state. Conformational clusters with a collapsed PLGA block (clusters 2 and 3) are more commonly found closer to the core–shell interface. Regarding the PEG block, one of the conformations has an extended PEG block (cluster 2) and the other one (cluster 3) has a collapsed PEG block. In a previous study, we investigated the dynamics of PEG blocks at the interface of polymeric NPs, showing that they exist in highly extended conformations as well as lie along the interface of the NP.^37^ The two conformations of these PEG–PLGA block copolymers identified at the core–corona interface, which are present in almost equal measures, are consistent with the contracted and extended states of the PEG blocks previously observed in those atomistic simulations. Therefore, the polymers take specific conformations as a result of their location within the NP, as was previously reported in our atomistic-level studies, where we have shown that block copolymers adopt location-specific conformations within micelles of various polymer chemistries and topologies.^37,40^

**Figure 3:**
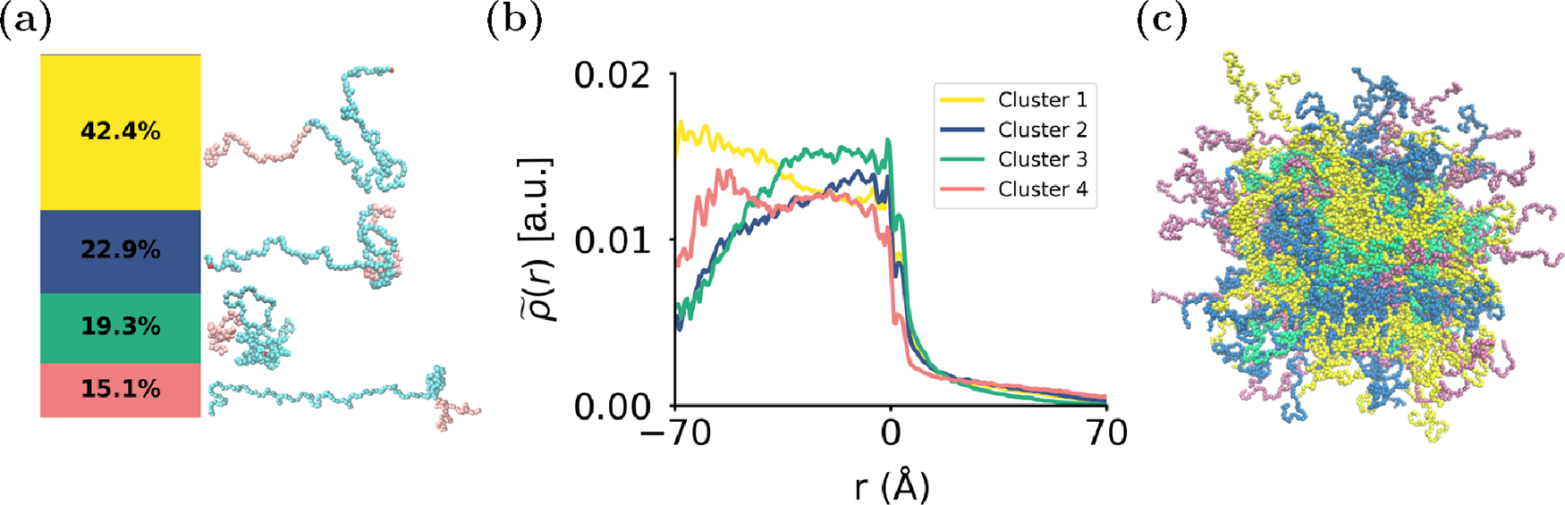
Unsupervised learning reveals location-specific polymer conformations. (a) Bar chart with the percentage of each cluster of polymer conformations within the NP with corresponding snapshots of a random polymer within each cluster. In the snapshots, PLGA is shown in pink and PEG in cyan. (b) Normalized intrinsic density profile of the various clusters within the NP. The normalization takes into account the cluster population. Normalized per polymer cluster. (c) Snapshot of NP with the polymers colored in correspondence to their cluster. The colors applied in the snapshot are the same as those used for the different clusters in (a) and (b). Polymer and NP snapshots are not to scale.

Having established this link between the location and polymer conformation, we investigated how EEK interacts with the different conformational states of the polymers in both the center of the core and at the core–corona interface. We calculated the number of contacts between each EEK peptide and each of the different polymer conformations, taking into account the relative abundance of polymer conformations in the two solubilization locations. This is essential in order to determine whether any preferential interactions exist between specific polymer conformations and EEK, rather than just local enrichment of a specific conformation in certain local environments of the NP (details of these computations can be found in the SI). We define the fractional enrichment, ϵ_*i*_, as follows
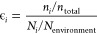
1where *n*_*i*_ is the number of contacts between the peptides and the polymers in conformational cluster *i* in its local environment (core or core–shell interface); *n*_total_ is the total number of contacts between the peptides and all polymers in the local environment. *N*_*i*_ is the number of polymers in conformational cluster *i* found in the local environment, and *N*_environment_ is the total number of polymers in the local environment. The fractional enrichment of polymer conformations quantifies the tendency of EEK to interact with a specific polymer cluster, taking into account the abundance of that polymer in the region of the NP where the EEK molecule is located. EEK peptides at the core–shell interface do not preferentially interact with a specific polymer conformation (Figure 4(a)); however, Figure 4(b) shows that there is a specific polymer environment that EEK peptides preferentially interact with in the hydrophobic core (cluster 4). EEK molecules found in both locations reside at an interface with water, interacting with the polymers at the point where the LA/GA and EO blocks join. Figures S9 and S10 show the normalized contact maps for peptides in the core and at the surface of the core of the NP, respectively. Peptides in both locations interact more with the EO block. Figure S9 shows that peptides within the core of the NP primarily interact with the final monomers of the GA block and the first few monomers of the EO block of cluster 4 polymers. While interacting with EO, EEK is also in close proximity to the hydrophobic PLGA block, reflecting EEK’s amphiphilicity. The polymers in cluster 4 are found near the trapped water interface: its extended EO block shields the more hydrophobic polymer blocks from water encapsulated in the NP core. Conversely, EEK at the core–corona interface generally interacts with the EO monomers of cluster 3 polymers and has very few contacts with the hydrophobic monomers, as shown in Figure S10.

**Figure 4:**
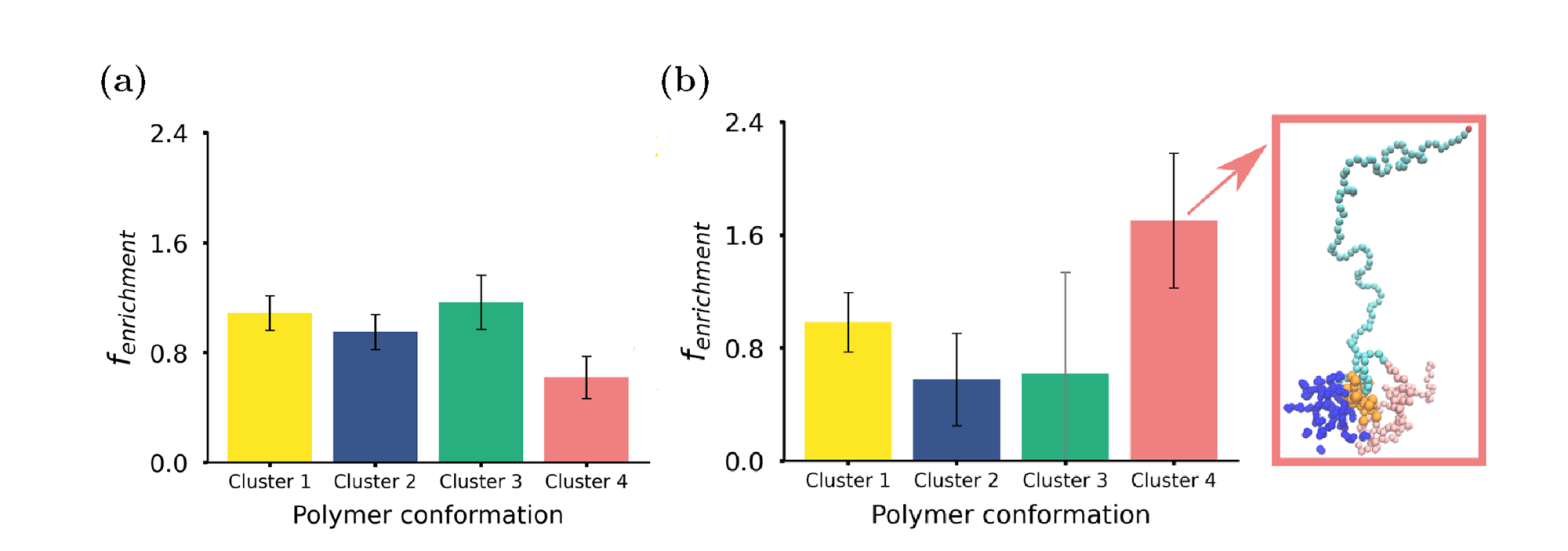
Cargo interactions with polymer conformational clusters. Average enrichment fraction of contacts between polymers in each of the different polymer clusters within the NP and (a) peptides at the core–shell interface and (b) peptides inside the core. Note that all error bars show the 90% CI. The enrichment takes into account the relative cluster population, such that an enrichment value greater than 1 means the peptides have a tendency to interact with that polymer conformation, an enrichment value of less than 1 suggests that there is a depletion of the polymers in that cluster around the peptide, and a value of 1 means that the peptide and the polymers from that cluster interact randomly. In (b), there is a snapshot showing a peptide interacting with the most preferential polymer cluster, cluster 4. In this snapshot, PLGA is colored pink, PEG is light blue, the peptide is orange, and water is navy blue. The error bar of cluster 3 in (b) is colored in gray due to poor statistics for this cluster, as there are not many interactions between the peptides captured inside the core and this cluster. The theoretical background for this calculation can be found in the SI.

In this work, we have integrated molecular-scale computer simulations and unsupervised machine learning techniques to demonstrate that therapeutic peptide solubilization by a polymeric NP does not depend solely on the overall NP structure but also depends on the specific conformations adopted by the individual polymers within it. These distinct conformations impart different local chemical environments that regulate drug encapsulation. We previously demonstrated that polymer topology controls the ability of polymers to adopt specific conformations within NPs upon their self-assembly.^40^ Together, these results suggest that the location-specific solubilization of drugs may be achieved by considering polymer topology and the resultant distribution of conformational states within self-assembled NPs.

## Supporting Information Available

(i) A detailed description of the analysis carried out, (ii) plots of the fraction of PLGA monomers in the core to analyze the equilibration time of the nanoparticle as a function of time, (iii) plots of the *R*_*G*_ of the nanoparticle as a function of time, (iv) the distance between the peptides and the COM of the nanoparticle, (v) the autocorrelation of the peptide local environment, (vi) the UMAP embedding and average cluster distances, (vii) the polymer cluster percentage and polymer cluster enrichment in the two storage locations of the peptides, and (viii) the normalized contacts between the peptides and the polymer cluster with which they interact the most (PDF)

## References

[ref1] Parveen S.; Sahoo S. K. Polymeric nanoparticles for cancer therapy. J. Drug Target. 2008, 16, 108–123. 10.1080/10611860701794353. 18274932

[ref2] Masood F. Polymeric nanoparticles for targeted drug delivery system for cancer therapy. Mater. Sci. Eng. 2016, 60, 569–578. 10.1016/j.msec.2015.11.067. 26706565

[ref3] Wissing S.; Kayser O.; Müller R. Solid lipid nanoparticles for parenteral drug delivery. Adv. Drug Delivery Rev. 2004, 56, 1257–1272. 10.1016/j.addr.2003.12.002. 15109768

[ref4] Torchilin V. P. Recent advances with liposomes as pharmaceutical carriers. Nat. Rev. Drug Discovery 2005, 4, 145–160. 10.1038/nrd1632. 15688077

[ref5] Joseph A.; Nyambura C. W.; Bondurant D.; Corry K.; Beebout D.; Wood T. R.; Pfaendtner J.; Nance E. Formulation and efficacy of catalase-loaded nanoparticles for the treatment of neonatal hypoxic-ischemic encephalopathy. Pharmaceutics 2021, 13, 1131 10.3390/pharmaceutics13081131. 34452092 PMC8400001

[ref6] Wang H.; Zhao Y.; Wu Y.; Hu Y.-l.; Nan K.; Nie G.; Chen H. Enhanced anti-tumor efficacy by co-delivery of doxorubicin and paclitaxel with amphiphilic methoxy PEG-PLGA copolymer nanoparticles. Biomater 2011, 32, 8281–8290. 10.1016/j.biomaterials.2011.07.032. 21807411

[ref7] Enlow E. M.; Luft J. C.; Napier M. E.; DeSimone J. M. Potent engineered PLGA nanoparticles by virtue of exceptionally high chemotherapeutic loadings. Nano Lett. 2011, 11, 808–813. 10.1021/nl104117p. 21265552 PMC3122105

[ref8] Wang Y.; Liu P.; Duan Y.; Yin X.; Wang Q.; Liu X.; Wang X.; Zhou J.; Wang W.; Qiu L.; et al. Specific cell targeting with APRPG conjugated PEG–PLGA nanoparticles for treating ovarian cancer. Biomater 2014, 35, 983–992. 10.1016/j.biomaterials.2013.09.062. 24176193

[ref9] Guo J.; Gao X.; Su L.; Xia H.; Gu G.; Pang Z.; Jiang X.; Yao L.; Chen J.; Chen H. Aptamer-functionalized PEG–PLGA nanoparticles for enhanced anti-glioma drug delivery. Biomater 2011, 32, 8010–8020. 10.1016/j.biomaterials.2011.07.004. 21788069

[ref10] Alibolandi M.; Sadeghi F.; Abnous K.; Atyabi F.; Ramezani M.; Hadizadeh F. The chemotherapeutic potential of doxorubicin-loaded PEG-b-PLGA nanopolymersomes in mouse breast cancer model. Eur. J. Pharm. Biopharm. 2015, 94, 521–531. 10.1016/j.ejpb.2015.07.005. 26170161

[ref11] Nyambura C. W.; Sampath J.; Nance E.; Pfaendtner J. Exploring structure and dynamics of the polylactic-co-glycolic acid–polyethylene glycol copolymer and its homopolymer constituents in various solvents using all-atom molecular dynamics. J. Appl. Polym. Sci. 2022, 139, e52732 10.1002/app.52732.

[ref12] Stipa P.; Marano S.; Galeazzi R.; Minnelli C.; Mobbili G.; Laudadio E. Prediction of drug-carrier interactions of PLA and PLGA drug-loaded nanoparticles by molecular dynamics simulations. Eur. Polym. J. 2021, 147, 110292 10.1016/j.eurpolymj.2021.110292. PMC8332736 34366437

[ref13] Jafari M.; Doustdar F.; Mehrnejad F. Molecular self-assembly strategy for encapsulation of an amphipathic α-helical antimicrobial peptide into the different polymeric and copolymeric nanoparticles. J. Chem. Inf. Model. 2019, 59, 550–563. 10.1021/acs.jcim.8b00641. 30475620

[ref14] Lee H. Molecular simulations of PEGylated biomolecules, liposomes, and nanoparticles for drug delivery applications. Pharmaceutics 2020, 12, 533 10.3390/pharmaceutics12060533. 32531886 PMC7355693

[ref15] Li Y.-P.; Pei Y.-Y.; Zhang X.-Y.; Gu Z.-H.; Zhou Z.-H.; Yuan W.-F.; Zhou J.-J.; Zhu J.-H.; Gao X.-J. PEGylated PLGA nanoparticles as protein carriers: synthesis, preparation and biodistribution in rats. J. Controlled Release 2001, 71, 203–211. 10.1016/S0168-3659(01)00218-8. 11274752

[ref16] Acharya S.; Sahoo S. K. PLGA nanoparticles containing various anticancer agents and tumour delivery by EPR effect. Adv. Drug Delivery Rev. 2011, 63, 170–183. 10.1016/j.addr.2010.10.008. 20965219

[ref17] Wilkosz N.; Łazarski G.; Kovacik L.; Gargas P.; Nowakowska M.; Jamroz D.; Kepczynski M. Molecular insight into drug-loading capacity of PEG–PLGA nanoparticles for itraconazole. J. Phys. Chem. B 2018, 122, 7080–7090. 10.1021/acs.jpcb.8b03742. 29927603

[ref18] Wilhelm S.; Tavares A. J.; Dai Q.; Ohta S.; Audet J.; Dvorak H. F.; Chan W. C. Analysis of nanoparticle delivery to tumours. Nat. Rev. Mater. 2016, 1, 16014 10.1038/natrevmats.2016.14.

[ref19] Zhu C.; Zhou X.; Liu Z.; Chen H.; Wu H.; Yang X.; Zhu X.; Ma J.; Dong H. The morphology of hydroxyapatite nanoparticles regulates cargo recognition in clathrin-mediated endocytosis. Front. Mol. Biosci. 2021, 8, 627015 10.3389/fmolb.2021.627015. 33748189 PMC7969717

[ref20] Asadzadeh H.; Moosavi A. Investigation of the interactions between Melittin and the PLGA and PLA polymers: molecular dynamic simulation and binding free energy calculation. Mater. Res. Express 2019, 6, 055318 10.1088/2053-1591/ab06d3.

[ref21] Asadzadeh H.; Moosavi A.; Arghavani J. The effect of chitosan and PEG polymers on stabilization of GF-17 structure: a molecular dynamics study. Carbohydr. Polym. 2020, 237, 116124 10.1016/j.carbpol.2020.116124. 32241401

[ref22] Chen C. H.; Liu Y.-H.; Eskandari A.; Ghimire J.; Lin L. C.-W.; Fang Z.-S.; Wimley W. C.; Ulmschneider J. P.; Suntharalingam K.; Hu C.-M. J.; et al. Integrated Design of a Membrane-Lytic Peptide-Based Intravenous Nanotherapeutic Suppresses Triple-Negative Breast Cancer. Adv. Sci. 2022, 9, 2105506 10.1002/advs.202105506. PMC9069370 35246961

[ref23] Berendsen H. J.; van der Spoel D.; van Drunen R. GROMACS: a message-passing parallel molecular dynamics implementation. Comput. Phys. Commun. 1995, 91, 43–56. 10.1016/0010-4655(95)00042-E.

[ref24] Marrink S. J.; Risselada H. J.; Yefimov S.; Tieleman D. P.; De Vries A. H. The MARTINI force field: coarse grained model for biomolecular simulations. J. Phys. Chem. B 2007, 111, 7812–7824. 10.1021/jp071097f. 17569554

[ref25] Jiao F.; Sang J.; Liu Z.; Liu W.; Liang W. Effect of concentration of PEG coated gold nanoparticle on lung surfactant studied with coarse-grained molecular dynamics simulations. Biophys. Chem. 2020, 266, 106457 10.1016/j.bpc.2020.106457. 32890945

[ref26] Pannuzzo M.; Esposito S.; Wu L.-P.; Key J.; Aryal S.; Celia C.; Di Marzio L.; Moghimi S. M.; Decuzzi P. Overcoming nanoparticle-mediated complement activation by surface PEG pairing. Nano Lett. 2020, 20, 4312–4321. 10.1021/acs.nanolett.0c01011. 32259451

[ref27] Pannuzzo M.; Felici A.; Decuzzi P. a coarse-grained molecular dynamics description of docetaxel-conjugate release from plga matrices. Biomacromolecules 2022, 23, 4678–4686. 10.1021/acs.biomac.2c00903. 36237166 PMC9667470

[ref28] Monticelli L.; Kandasamy S. K.; Periole X.; Larson R. G.; Tieleman D. P.; Marrink S.-J. The MARTINI coarse-grained force field: extension to proteins. J. Chem. Theory Comput. 2008, 4, 819–834. 10.1021/ct700324x. 26621095

[ref29] Yesylevskyy S. O.; Schäfer L. V.; Sengupta D.; Marrink S. J. Polarizable water model for the coarse-grained MARTINI force field. PLoS Comput. Biol. 2010, 6, e1000810 10.1371/journal.pcbi.1000810. 20548957 PMC2883601

[ref30] Hinkle K. R. Using coarse-grained models to examine structure-property relationships of diblock-arm star polymers. Eur. Polym. J. 2021, 142, 110149 10.1016/j.eurpolymj.2020.110149.

[ref31] Grunewald F.; Rossi G.; de Vries A. H.; Marrink S. J.; Monticelli L. Transferable MARTINI model of poly (ethylene oxide). J. Phys. Chem. B 2018, 122, 7436–7449. 10.1021/acs.jpcb.8b04760. 29966087

[ref32] Jo S.; Kim T.; Iyer V. G.; Im W. CHARMM-GUI: a web-based graphical user interface for CHARMM. J. Comput. Chem. 2008, 29, 1859–1865. 10.1002/jcc.20945. 18351591

[ref33] Qi Y.; Ingólfsson H. I.; Cheng X.; Lee J.; Marrink S. J.; Im W. CHARMM-GUI Martini maker for coarse-grained simulations with the Martini force field. J. Chem. Theory Comput. 2015, 11, 4486–4494. 10.1021/acs.jctc.5b00513. 26575938

[ref34] Hess B.; Kutzner C.; Van Der Spoel D.; Lindahl E. GROMACS 4: algorithms for highly efficient, load-balanced, and scalable molecular simulation. J. Chem. Theory Comput. 2008, 4, 435–447. 10.1021/ct700301q. 26620784

[ref35] Bussi G.; Donadio D.; Parrinello M. Canonical sampling through velocity rescaling. J. Chem. Phys. 2007, 126 (1), 014101 10.1063/1.2408420. 17212484

[ref36] Bjelkmar P.; Larsson P.; Cuendet M. A.; Hess B.; Lindahl E. Implementation of the CHARMM force field in GROMACS: analysis of protein stability effects from correction maps, virtual interaction sites, and water models. J. Chem. Theory Comput. 2010, 6, 459–466. 10.1021/ct900549r. 26617301

[ref37] Ziolek R. M.; Smith P.; Pink D. L.; Dreiss C. A.; Lorenz C. D. Unsupervised learning unravels the structure of four-arm and linear block copolymer micelles. Macromolecules 2021, 54, 3755–3768. 10.1021/acs.macromol.0c02523.

[ref38] McInnes L.; Healy J.; Melville J. Umap: Uniform manifold approximation and projection for dimension reduction. arXiv 2018, 10.48550/arXiv.1802.03426 (accessed on 2023-02-03).

[ref39] McInnes L.; Healy J.; Astels S. Hierarchical density based clustering. J. Open Source Softw. 2017, 2, 205 10.21105/joss.00205.

[ref40] Lopez-Rios de Castro R.; Ziolek R. M.; Lorenz C. D. Topology-controlled self-assembly of amphiphilic block copolymers. Nanoscale 2023, 15, 15230–15237. 10.1039/D3NR01204B. 37671739 PMC10540979

[ref41] King’s College London. King’s Computational Research, Engineering and Technology Environment (CREATE) 2022, 10.18742/rnvf-m076.

