# Supplementary material for: Therapeutic Peptides Are Preferentially Solubilized in Specific Microenvironments within PEG–PLGA Polymer Nanoparticles
Source: Nano Lett. 2024 Feb 2;24(6):2011–7. doi: 10.1021/acs.nanolett.3c04558 (PMC10870757; doi:10.1021/acs.nanolett.3c04558)
Supplement: Supplementary file 1 — nl3c04558_si_001.pdf [file nl3c04558_si_001.pdf]

Supplementary Information for "Therapeutic peptides are preferentially solubilized in specific microenvironments within PEG-PLGA polymer nanoparticles"

Raquel López-Rios de Castro,[†,‡] Robert M. Ziolek,[‡] Martin B. Ulmschneider,[†] and Christian D. Lorenz[*,‡]

†*Department of Chemistry, King's College London, London, SE1 1DB, United Kingdom*

‡*Biological Physics and Soft Matter Group, Department of Physics, King's College London, London, WC2R 2LS, United Kingdom*

E-mail: chris.lorenz@kcl.ac.uk

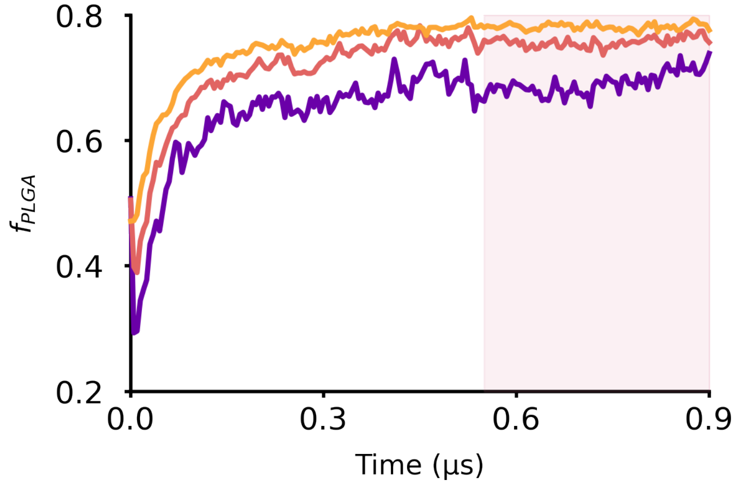

Figure S1: **Micelle structural equilibration analysis.** Fraction of PLGA monomers in the core as a function of time. Purple is a distance of $r < 30$ Å, red $r < 40$ Å, and orange $r < 50$ Å from the micelle center of mass. The area shaded in pink denotes the time from which the nanoparticle is determined to have reached structural equilibration.

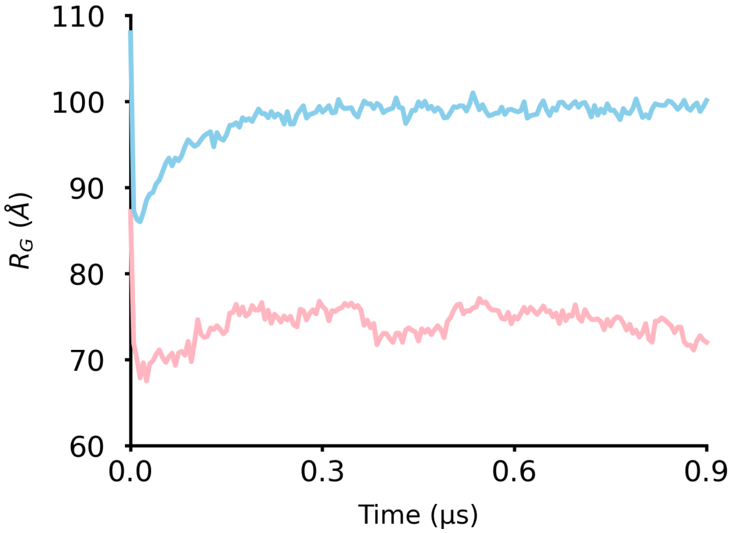

Figure S2: **Nanoparticle radius of gyration over time.** The radius of gyration of the nanoparticle core is shown in pink and that of the whole nanoparticle is shown in cyan. Note that these quantities appear to reach stationarity more quickly than the distribution of PLGA within the NP as reported Figure S1.

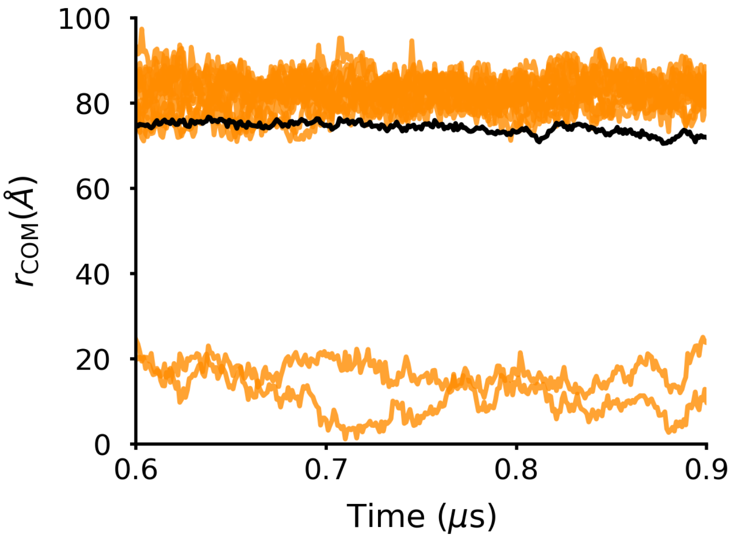

Figure S3: **Cargo distance to nanoparticle's center of mass.** Distance from all peptides to the center of mass of the nanoparticle over time. Orange lines denote each of the peptides and the black line is the R_g of the core. It is clear that there are two storage locations, one very close to the center, populated by two peptides, and another one at the core-shell interface with the remaining peptides.

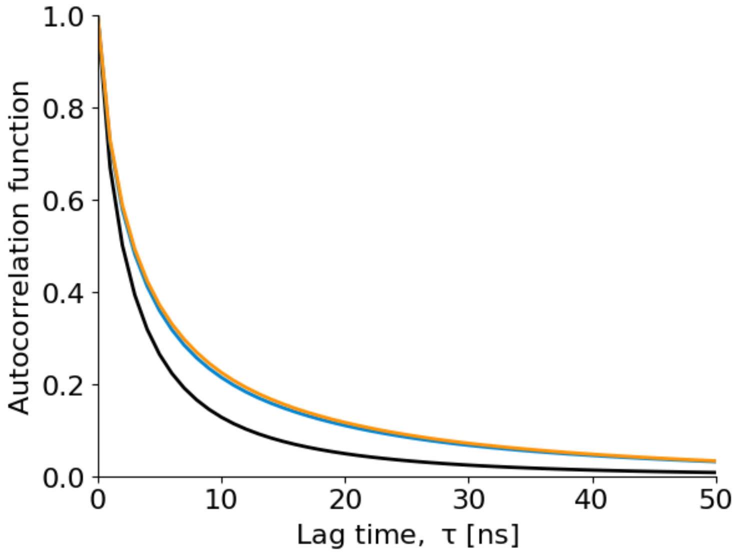

Figure S4: **Autocorrelation of the peptide local environment.** The autocorrelation functions show the change in local environment of the peptides (average of peptides at the core-corona interface are shown in black and the two peptides found in the micelle core are shown in blue and orange). The peptide local environment is defined as all polymer beads found within 7.5 Å of any bead that is part of a specific peptide.

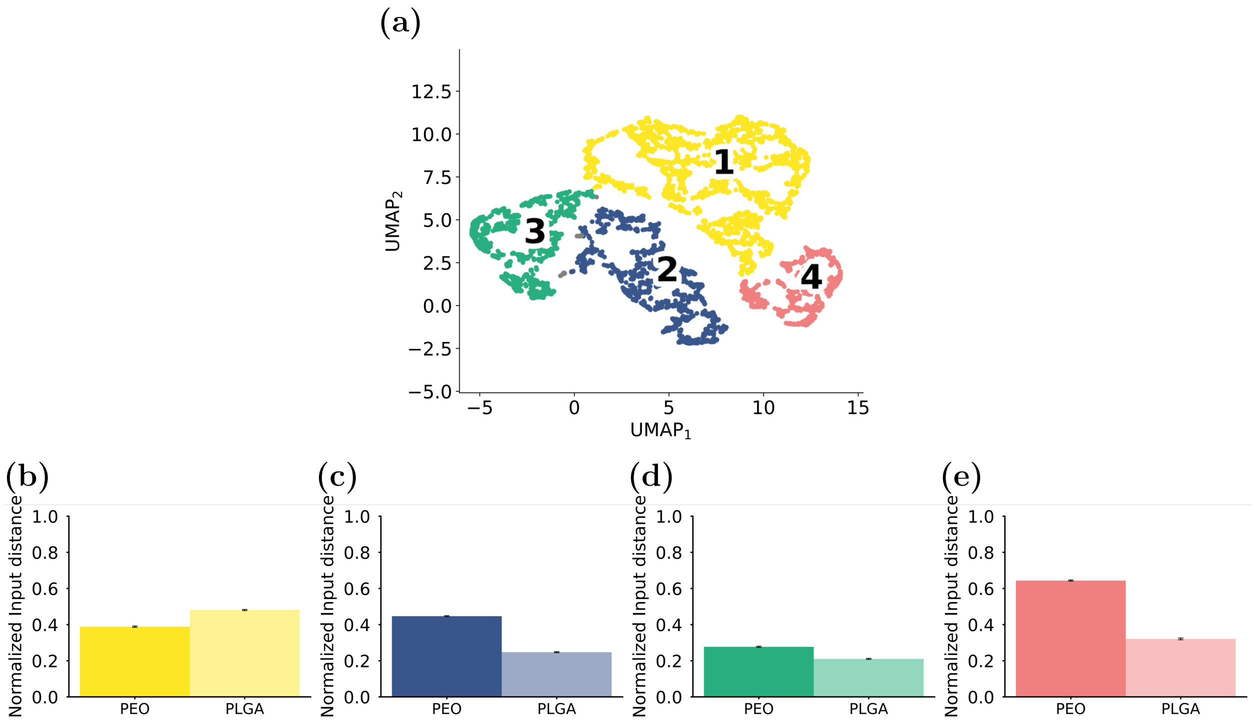

Figure S5: **UMAP embedded space and average cluster distances.** (a) UMAP embedded space clustered by HDBSCAN of polymers. Histograms of the average distances of each cluster: (a) cluster 1, (b) cluster 2, (c) cluster 3 and (d) cluster 4. Note that the error bars show the 90% CI.

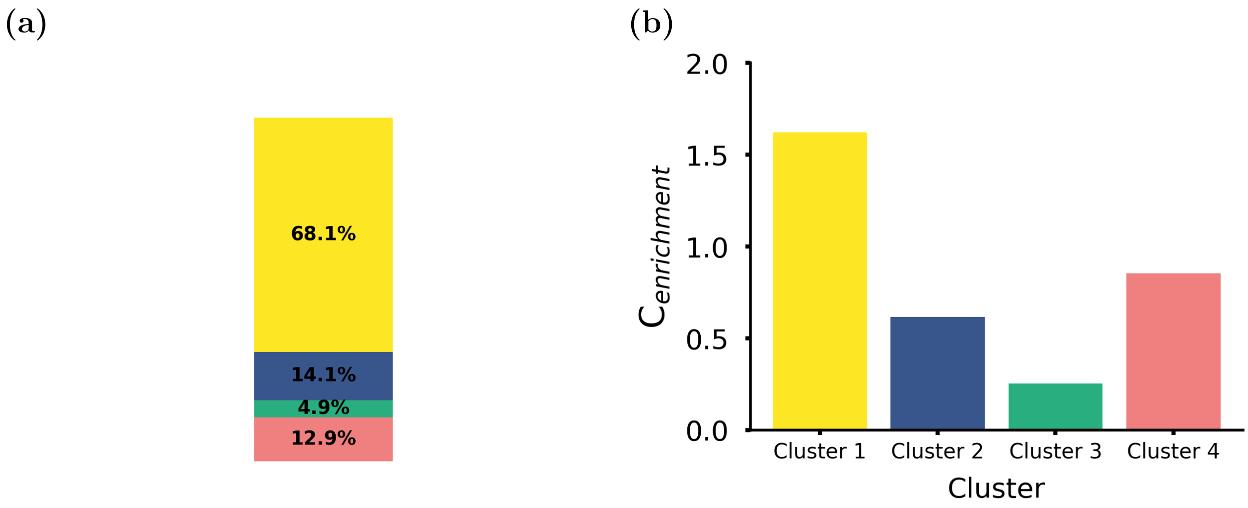

Figure S6: **Polymer micro-environment of the inner storage location.** (a) The percentage of polymers near the peptides in the core of the NP that are in each conformational cluster. (b) Enrichment of the polymers within each of the conformational clusters near the peptides in the core of the NP. A value greater than 1 means that the cluster is more present in this region than it is throughout the entire NP, and a value smaller than 1 means the cluster is depleted in that area.

Contacts calculation. Contacts are used in this manuscript to study the interactions between the protein, polymer and water. The contacts reported in Figure 2 are calculated by computing the distance between the protein center of mass and all polymer monomer positions. If this distance was below 6.5 Å, it is counted as a contact. This is repeated for all time steps, and the final values used for the difference calculation in Figure 2 are the time averages. For the hydration shown in Figure 2, a similar strategy was followed, the distance between the protein and water heavy atoms is calculated. If the distance is below 6.5 Å, then it is counted as a contact. Again, the value used for the difference calculation in Figure 2 are the time averages. The cutoff distances were obtained from the first peak of the radial distribution functions.

Contact enrichment between proteins and polymer species. Contact enrichment is a quantitative measure used to assess the preferential interaction between distinct molecular species within a given system, as revealed by molecular dynamics simulations. It quantifies the extent to which one molecule exhibits a higher tendency to interact with another

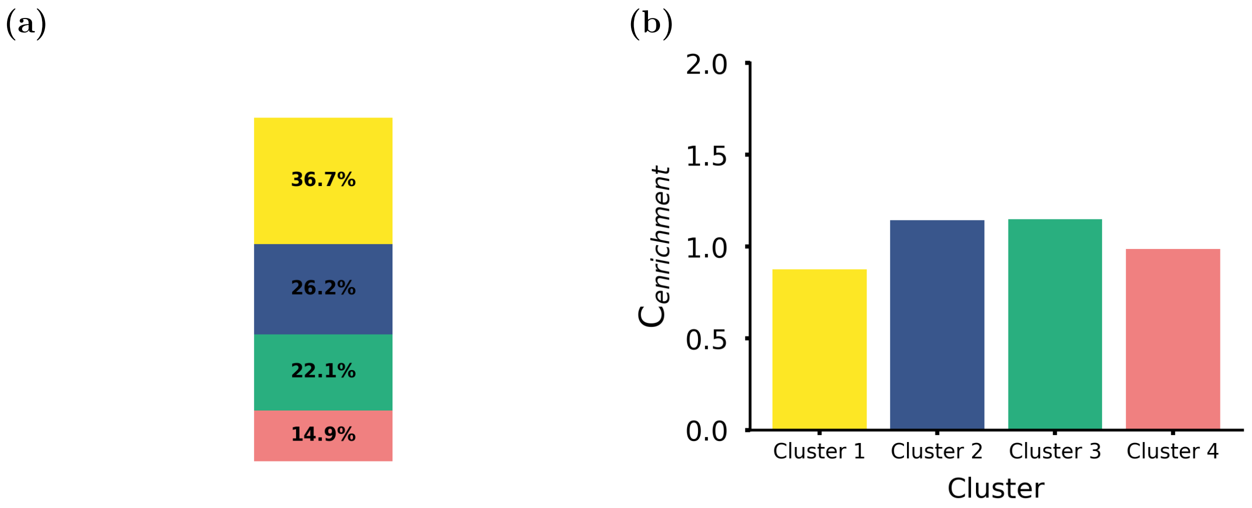

Figure S7: **Polymer micro-environment of core-shell interface storage location.** (a) Percentage of presence of clusters in the core-shell interface location of peptide storage. (b) Cluster presence enrichment of polymer clusters at the core-shell location of peptide storage with respect to overall cluster presence. A value greater than 1 means that the cluster is enriched in that region of the nanoparticle, and a value smaller than 1 means the cluster is underrepresented in that area.

molecule, relative to their respective abundances. This metric is computed by comparing the observed interaction frequency involving a specific molecule to the expected average interaction frequency, considering the relative proportions of the molecules involved. This contact enrichment $N_{\mathrm{enrichment}}$ is calculated with the following formula:

$$N_{\mathrm{enrichment}} = \frac{N_{\mathrm{pol\_species}}/N_{\mathrm{tot\_pol}}}{P_{\mathrm{pol\_species}}/P_{\mathrm{tot\_pol}}} \tag{1}$$

where $N_{\mathrm{pol\_species}}$ is the number of contacts between a protein and a specific polymer species, $N_{\mathrm{tot\_pol}}$ are the total contacts between a protein and all polymers. $P_{\mathrm{pol\_species}}$ are the number of monomers belonging to that specific polymer species and $P_{\mathrm{tot\_pol}}$ are the total number of all polymers. A contact between the protein and a polymer monomer was considered if any protein atom was within 6 Å of any polymer monomer. This was calculated at every time step and the values reported in Table 1 are the average over time for the two separate storage locations.

Intrinsic Core-Shell Interface (ICSI) Method. For micelles with an irregular inter-

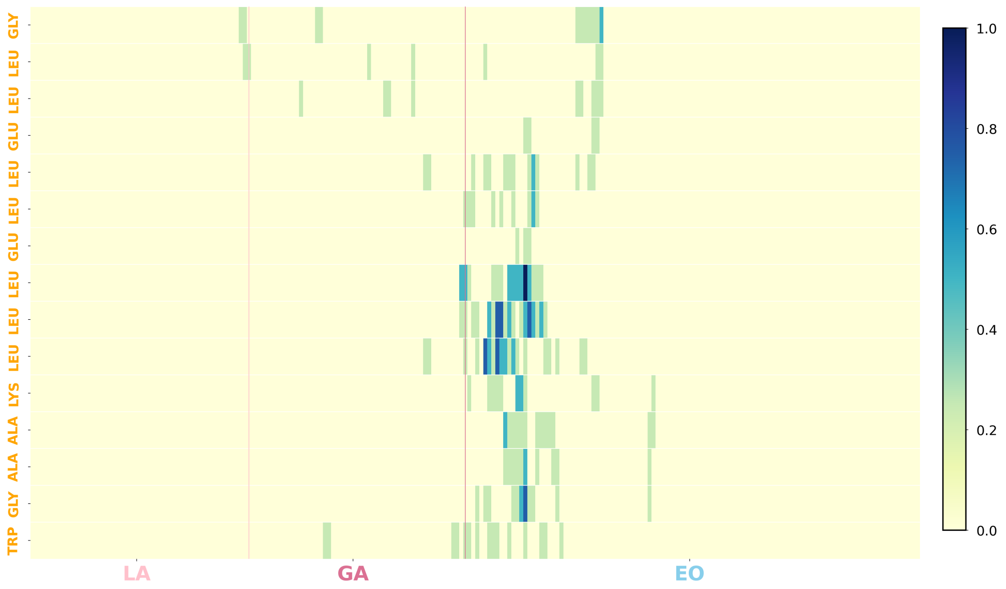

Figure S8: **Normalized contacts between peptides in the inner location and polymer conformation cluster 4.** Normalized contacts between peptides in the inner storage locations and cluster 4 polymer species over time.

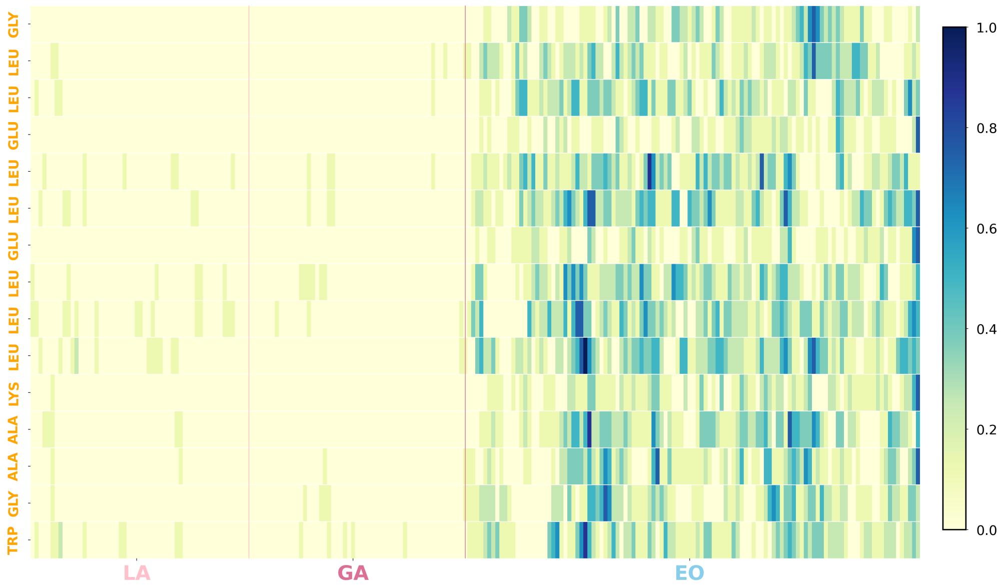

Figure S9: **Normalized contacts between peptides at the core-shell interface location and polymer conformation cluster 3.** Normalized contacts between peptides at the core-shell interface storage locations and cluster 3 polymer species over time.

facial structure, intrinsic interface techniques can be used to investigate their internal and interfacial structure.[1] For this method we selected the MA heavy atoms to form the core of the micelle, as as they are the principal component of the core (this information could be inferred by the contact maps, the hydration data and the spherical density of components). The grid selected was 21 × 21. Detailed information on the working of this algorithm was first presented by Ziolek et al.[1] The ICSI equation is:

$$\tilde{\rho}(r) \equiv \left\langle \sum_i \frac{\delta[r - (r_i - \xi(\theta, \phi))]}{\bar{S}_i(r)} \right\rangle \tag{2}$$

where r_i is the r-position of atom i (of the chosen group of atoms) and $\xi(\theta, \phi)$ is the r-position of the ICSI. The average volume of the shell in which a given atom is found when using the intrinsic surface approach, $\bar{S}_i(r)$, which normalizes the intrinsic density, is given by:

$$\bar{S}_i(r) = \frac{n_i \bar{V}_{\text{box}}}{N} \tag{3}$$

where n_i is the number of points found in the shell in which atom i is found over all the clusters analyzed, $\bar{V}_{\text{box}}$ is the average volume of the simulation box, and N is the total number of random coordinates used in the normalization procedure.

Dimensionality reduction and clustering. The distances chosen as the input space to generate the two dimensional UMAP embedded data are topology specific. The goal was to find the minimum number of distances that could represent the conformational complexity adopted by the polymers. Two distances were selected, the distance between the terminal monomers of the PLGA block and the distance from the terminal monomers of the PEO block. These two distances were sufficient to capture the complexity of the polymer conformations. The UMAP embedded output was later clustered with HDBSCAN, Table S1 shows the UMAP and HDBSCAN parameters chosen.

The intrinsic density of the UMAP clusters was calculated in the same way as for the overall micelle intrinsic density. But instead of using all polymers, only the polymers be-

longing to the specific cluster whose density was being calculated were used.

Table S1: UMAP (*n_neighbours*) and HDBSCAN (*min_cluster_size* and *cluster_selection_epsilon*) parameters

	Parameters
n_neighbours	25
min_cluster_size	45
cluster_selection_epsilon	0.85

Cargo interactions with polymer clusters. After obtaining the polymer cluster conformations, we looked into how proteins interact with the different clusters. For this we first calculated the enrichment of the different polymer conformational clusters near the two different peptide storage locations. In order to characterise the local environment in the core of the NP near where the peptides are stored we considered all polymers that were within a radius of 25 Å from the center of mass of the NP. For the core/corona interface environment, we considered the polymers that were found between 65 Å and 90 Å from the center of mass of the micelle. Then we determined the enrichment of the clusters, $C_{i,\text{enrichment}}$, as follows:

$$C_{i,\text{enrichment}} = \frac{N_{i,\text{local}}/N_{\text{tot,local}}}{N_i/N_{\text{total}}} \tag{4}$$

where $N_{i,\text{local}}$ is the number of polymers from cluster i in the local environment, $N_{\text{tot,local}}$ is the total number of polymers in the local environment, N_i is the number of polymers from cluster i in the entire NP and N_{total} is the total number of polymers in the entire NP. Figures S6(a) and S7(a) show the values of $C_{i,\text{enrichment}}$ for the core and core/corona interface environments, respectively. Then, the contacts between the peptides and the polymers in these environments were calculated. A contact was considered if the center of mass of a protein was within 6.5 Å of any polymer bead. This was calculated at every time step. Then the cluster labels of each polymer in contact with the protein were identified, and the number of contacts with each cluster was counted and averaged over the peptides in the different

environments and over time.

Confidence interval calculation. Error bars in the histograms of this manuscript are the confidence intervals calculated at a 90% confidence. They were calculated with the following formula:

$$CI = \bar{x} \pm z\frac{\sigma}{\sqrt{n}} \tag{5}$$

where $\bar{x}$ is the mean of the sample, z is set to 1.645 for a 90% CI, σ is the standard deviation and n is the sample size.

References

(1) Ziolek, R. M.; Smith, P.; Pink, D. L.; Dreiss, C. A.; Lorenz, C. D. Unsupervised learning unravels the structure of four-arm and linear block copolymer micelles. *Macromolecules* **2021**, *54*, 3755–3768.
